# Supplementary material for: In vivo semen characterization and seasonal variation in Procavia capensis
Source: Reprod Fertil. 2026 Jan 12;7(1):RAF250132. doi: 10.1530/RAF-25-0132 (PMC13138600; doi:10.1530/RAF-25-0132)
Supplement: Supplementary file 1 [file supplementary_materials.pdf]

In vivo semen characterization and seasonal variation in *Procavia capensis*

Tal Raz[1,2,*], Hunter Warick[3], Stav Asulin-Schnaiderman[1], Nuphar Shidlovsky[3], Nathalie Weizmann[1], Lee Koren[3]

[1] Koret School of Veterinary Medicine, Robert H. Smith Faculty of Agriculture, Food and Environment, The Hebrew University of Jerusalem, Rehovot, Israel;

[2] Advanced Academic Programs, Krieger School of Arts and Sciences, Johns Hopkins University, Baltimore, MD, USA.

[3] Faculty of Life Sciences, Bar Ilan University, Ramat Gan, Israel.

*Correspondence: Tal.Raz@mail.huji.ac.il

Table S1. Summary of semen collection attempts and responses to electroejaculation in captive rock hyrax males (September 2017 - October 2018).

No.	Date	Male ID	BW (kg)	Erection: Yes/No (stimulation parameters)	Ejaculation: Yes/No	Ejaculation: Physical appearance	Ejaculation: Presence of Sperm Cells (Yes/No)
1	17 Sep 2017	C3	3.3	Yes	Yes	Milky opaque	Yes
2	16 Nov 2017	C1	2.5	Yes	Yes	Clear	No (Only round cells)
3	16 Nov 2017	C2	2.6	Yes	No	---	---
4	28 Jan 2018	C1	2.6	Yes	No	---	---
5	28 Jan 2018	C2	2.6	Yes	Yes	Clear yellowish	No
6	28 Mar 2018	C1	3.1	Yes (within ~7 min)	No	---	---
7	28 Mar 2018	C2	2.7	Yes (within ~3 min)	No	---	---
8	29 Apr 2018	C1	3.2	Yes (within ~2.5 min)	Yes	Clear yellowish, then viscous	No
9	29 Apr 2018	C2	2.8	Yes (within ~2.5 min; at 4V)	Yes	Clear yellowish, then viscous	No
10	16 May 2018	C1	3.3	Yes (within ~2 min; at 4V)	Yes	Clear-milky	No (Only round cells)
11	16 May 2018	C2	2.8	Yes (within ~2 min; at 3V)	Yes	Clear-milky	No (Only round cells)
12	19 Jul 2018	C2	2.9	Yes (within ~3.5 min; at 3.5V)	Yes	Milky opaque	Yes
13	12 Aug 2018	C1	3.1	Yes (within ~8.5 min; at 8.5V)	Yes	Milky opaque	Yes
14	13 Sep 2018	C2	2.9	Yes (within ~6.5 min; at 7V)	Yes	Milky opaque	Yes
15	17 Sep 2018	C2	3	Yes (within ~2 min; at 3V)	Yes	Milky opaque	Yes
16	9 Oct 2018	C2	3.2	Yes (Partial) (within ~6.5 min; at 4V)	No	---	---
17	9 Oct 2018	C1	3.1	Yes (within ~6 min; at 6V)	Yes	Milky opaque	Yes
